# Repair of Proximal Hamstring Tear Utilizing a Suture Bridge Knotless Construct

**DOI:** 10.1155/2020/8840418

**Published:** 2020-08-02

**Authors:** Brent Sanderson, Kyle Stumetz, Reza Jazayeri

**Affiliations:** ^1^Community Memorial Health System-Department of Orthopaedic Surgery, 147 N. Brent St. Ventura, CA 93003, USA; ^2^Pacific Northwest University School of Medicine, Butler-Haney Hall, 200 University Pkwy, Yakima, WA 98901, USA; ^3^Kaiser Permanente of Southern California-Department of Orthopaedic Surgery, 888 S Hill Rd, Ventura, CA 93003, USA

## Abstract

Proximal hamstring tendon injuries occur frequently in the athletic population resulting in varying degrees of functional disability depending on severity of injury. The purpose of our case vignette is to describe a surgical technique and clinical outcome for open proximal hamstring tendon repair with a confirmed biomechanically sound construct. We also describe and summarize the current literature recommendations for proximal hamstring injuries. We present a case and surgical technique report on a 27-year-old male who suffered a proximal hamstring tendon rupture. Utilizing a double row all-knotless suture bridge construct with a total of four anchors and six suture limbs allowed for anatomic footprint coverage and strength. Two years of clinical follow-up was obtained evaluating hip and knee range of motion, strength, and functional ability. Our patient underwent uncomplicated open surgical repair and returned to all activity at four months following surgery. Range of motion and strength returned to preoperative levels at the four-month postoperative mark. The use of a reproducible double row all-knotless suture bridge technique provided adequate strength and stability in the setting of a proximal hamstring tendon rupture. Open and endoscopic surgical techniques performed acutely both show positive postoperative subjective outcomes as well as a high likelihood of returning to sport. Controversy remains present in regard to the repair technique as well as postoperative bracing and physical therapy recommendations.

## 1. Introduction

The proximal hamstring complex is an important group of synergistic muscles that function to extend the hip and flex the knee. It is composed of three distinct muscles including the long head of the biceps femoris, the semitendinous muscle, and semimembranous muscle. The biceps femoris and the semitendinous muscle form a conjoint tendon that inserts medially on the ischial tuberosity, while the semimembranosus muscle inserts laterally.

During eccentric contraction, there is substantial load placed upon these muscles predisposing them to increased strain and tearing during high-intensity activities. This risk is significantly increased in sports that require running, jumping, and kicking (such as hurdling, soccer, and American football) as well as during the initial sit to stand phase of waterskiing.

Hamstring injuries represent nearly 30% of new lower extremity complaints, with the hamstring muscle complex (HMC) being the most frequently injured. The vast majority of injuries occur at the myotendinous junction [1]. HMC injuries are typically treated conservatively with an initial period of protected weight bearing and rest, ice, compression, elevation (RICE) therapy, followed by strength and balancing exercises. Surgical intervention becomes necessary when the patient fails to improve with conservative management or meets specific surgical indications.

Patients commonly report a history of an acute, sharp pain felt just prior to the initiation of rapid acceleration or deceleration activities or the history of traumatic blow to the lower extremity while the patient's hip is fully flexed and the knee fully extended. Pain is most commonly centralized to the posterior thigh, with symptomatology over the ischial tuberosity being more suggestive of an avulsion injury, rather than a myotendinous injury. Oftentimes, an audible pop or the feeling of a snapping sensation can also be felt and, in severe cases, ecchymosis and a palpable mass may even be visualized on the posterior thigh due to proximal tendon avulsion and distal retraction [1].

Generally accepted indications for surgical repair include ischial tuberosity bony avulsion or tendon retraction with >2 cm displacement and more than one hamstring tendon involvement [2, 3]. Wood and colleagues [3] reviewed 72 hamstring repair cases and found that in cases of complete avulsion with hamstring retraction, a delay in surgical repair results in a more technically challenging repair, increasing the likelihood of sciatic nerve involvement, increasing the need for postoperative bracing, and reducing postoperative hamstring strength and endurance. Along with a delay in surgical repair, increased tendon retraction has been linked to worse functional outcomes [3]. Piposar and associates [2] performed a retrospective case series of 25 patients with hamstring injuries. They found that 40% of nonsurgically treated proximal hamstring avulsions of <2 cm eventually required surgical intervention. In patients with proximal hamstring injuries without significant retraction, <2 cm, an individualized treatment decision is recommended given the high propensity for need for surgical treatment in the future if the patient fails conservative therapy [1, 2].

Repair of the proximal hamstring can be performed either open or endoscopically and performed with a variety of fixation constructs based on the surgeon's preference. Management of proximal hamstring avulsion injuries can be challenging from both an operative and a rehabilitation standpoint. The purpose of this case report and review is to describe our preferred approach for open surgical repair and to further reinforce the utility of a knotless double row suture bridge repair in the setting of a retracted, triple tendon, proximal hamstring avulsion.

## 2. Case Report

Our patient is a 27-year-old healthy active individual with no past medical problems (see Supplemental Video 1). He felt sharp pain located in the left buttocks area and posterior thigh during a soccer game. He had immediate pain and inability to ambulate without limping. On a physical exam, the patient had an antalgic stiff-legged gait that minimized both hip and knee motion. Marked tenderness with palpation was present along the proximal hamstring muscle belly complex and ischial tuberosity. Left hip active range of motion was limited secondary to pain. Motor strength testing revealed significant weakness in hip extension and knee flexion compared to the contralateral side. The heel drag test elicited a positive result, with pain and weakness compared to the uninjured side.

**Figure 1:**
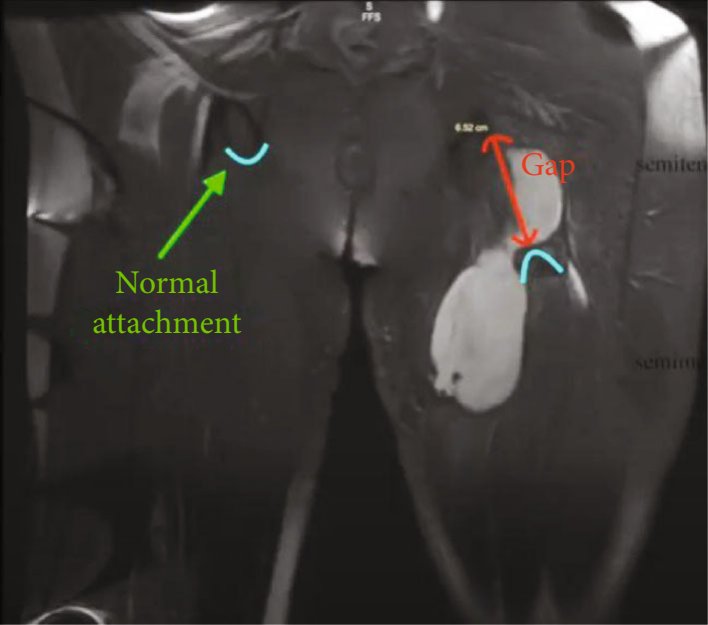
Coronal MRI displaying a retracted complete tear is of the left semitendinosus and biceps femoris tendons from the origin. Associated severe partial tear of the semimembranosus tendon with associated hematoma within the posterior deep musculature measuring up to 16.5 cm.

Magnetic resonance imaging (MRI) of the injured extremity demonstrated a left proximal complete tear of the left semitendinosus and biceps femoris tendons from the origin with retraction (see Figure 1). There was also associated severe partial tear of the semimembranosus tendon. Plain radiographs were negative for bony fracture or other abnormality.

The patient was employed as a manual laborer and was unable to return to work following his injury. Due to his continued discomfort, inability to perform his job, and multiple tendon involvement, surgical intervention was pursued 3 weeks following the injury.

## 3. Surgical Technique

**Figure 2:**
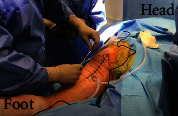
Intraoperative image of the planned longitudinal incision width.

Following verbal and written consent per protocol, the patient was brought into the operative arena. Intubation and general anesthesia was initiated while the patient was supine. The patient was then placed in the prone position. All bony and soft tissue prominences were well padded. Standard sterile preparation with chloraprep and draping was then performed. Surgical timeout was completed, and a longitudinal incision was made due to the chronicity of the tear and possible need for extensive dissection (see Figure 2). The superficial cutaneous nerves were protected throughout the course of the case. Once the fascia was incised, the gluteus medius was reflected superiorly. The hematoma from the hamstring rupture, approximately 6 cm distal to the ischial tuberosity, was identified and was consistent with the preoperative MRI findings. Extensive amount of scarring along the course of the sciatic nerve was noted. Neurolysis of the sciatic nerve was performed. Special care was taken to protect the sciatic nerve throughout the course of the case.

After the wound was copiously irrigated, a modified suture bridge repair was carried out. Initially, sutures were passed 1.5-2 cm into the stump of the retracted ruptured tendon to aid in visualization and lysis of tendon adhesion to surrounding tissue. The knee was bent to approximately 50 degrees in order to relieve tension of the repair. The ischial tuberosity was identified and retractors were placed to protect the sciatic nerve and expose the ischial tuberosity. The footprint for the insertion of the hamstring tendons was identified and debrided in order to provide a healthy bleeding bed for improved biological healing.

**Figure 3:**
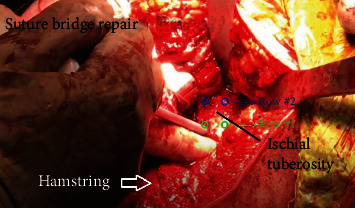
Intraoperative photo demonstrating placement of the first anchor of the planned double row repair into the ischial tuberosity hamstring footprint.

**Figure 4:**
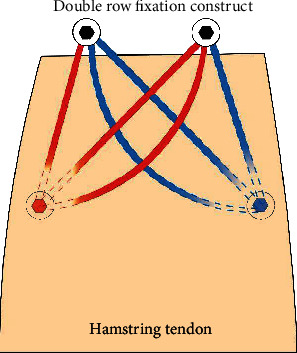
Double row construct. Blue: triple loaded suture anchor with three SutureTapes and red: triple loaded suture anchor with three SutureTapes.

Two 2.6 mm FiberTak Soft Anchor (Arthrex, Naples, FL), triple loaded with three 1.3 mm SutureTapes were placed at the distal aspect of the ischial footprint (see Figure 3). The anchors were placed with 8 mm of separation between each other. Once the anchors are secured, a free needle was utilized to pass the sutures sequentially through the tendon with full-thickness passes. Each free suture limb was passed from distal to proximal through the tendon with at least a 2 mm gap between each suture pass. The suture limbs were brought over proximally in order to create a suture bridge construct. The proximal row anchors were placed at the proximal aspect of the ischial footprint, one medial and one lateral, using 4.75 mm SwiveLock anchors (Arthrex, Naples, FL). A total of six suture limbs were placed into each eyelet of the SwiveLock anchor to create a knotless construct (see Figure 4). Appropriate tension was applied to the suture limbs to provide solid fixation of the tendon to its anatomic position. Probing demonstrated robust compression of the tendon back to its anatomic footprint.

The incision was then copiously irrigated. The knee was easily extended to 50 degrees without undue tension on the repair. At approximately 45 degrees of knee flexion, an increased amount of tension was noted at the repair site. Layered closure was completed in the usual fashion. The patient was placed in a locked hinged knee brace locked in 90 degrees.

Postoperatively, the patient was made non-weight bearing for a total of six weeks. He was instructed to use the hinged knee brace for the first six weeks with strict precautions on the locked position of 90 degrees for the first two weeks. After the initial two-week recovery, from postoperative weeks 2 to 4, the brace was set to allow 90 to 50 degrees of knee flexion. At four weeks following surgery, the patient gradually increased the amount of knee extension allowed by 10 to 15 degrees per week. No strengthening was allowed until eight weeks postoperatively. Concentric strengthening was permitted after 8 weeks, and eccentric strengthening was initiated 3 months postoperatively.

Two years of clinical follow-up was obtained evaluating hip and knee range of motion, strength, and functional ability. The patient demonstrated similar strength and range of motion compared to the contralateral nonoperative extremity. The patient returned to work 4 months following surgery without functional limitation.

## 4. Discussion

Increasing activity with a focus on cross-training exercises in the general population along with the chronic high-performance demands on athletes at all levels results in injuries in the HMC being more and more common. Although ruptures and strains can occur in all three hamstring muscles, the most commonly injured is the biceps femoris [4], with injuries more likely to occur proximally rather than distally. Despite the most meticulous rehabilitation, many cases are recalcitrant to nonsurgical intervention, and others with more severe presentations such as avulsions or retraction require early surgical management. These factors lead to increased athlete morbidity.

Ensuring strength and stability in a repair construct is of the utmost importance to allow for adequate time for tendon healing. A recent study confirmed hamstring healing capability following proximal repair. In the study, a postoperative MRI, completed at a mean of 36+/-11.4 months following repair, demonstrated in all 12 cases, the HMC reattached to the ischial tuberosity. The hamstring repair was completed with an average of 2.5 anchors and sutures passed and tied in a Mason-Allen–type fashion [5].

Multiple surgical techniques, both open and endoscopic, have been reported in the literature for proximal hamstring ruptures and ischial tuberosity bony avulsions. Suture anchor fixation has provided increased variability and versatility in proximal hamstring repair with less soft tissue disruption compared to transosseous fixation methods. In a recent case report, Tetsunaga et al. [6] describe successful surgical treatment of an avulsion fracture of the ischial tuberosity by suture anchor fixation using a suture bridge technique. Moatshe and colleagues [7] utilized their group's biomechanical cadaveric study to perform a five-suture anchor technique which was shown to be significantly stronger than repairs with two large or small anchors.

Minimally invasive endoscopic repair was described by Mehta et al. [8] as another alternative method to suture anchor fixation. Different patterns of suture anchor configuration have been employed, including five anchors in an “X” pattern as well as six anchors positioned like the face of a die. Additionally, luggage tag sutures at the medial leading edge of the tendon can be utilized to improve bony contact [1, 9].

Gerhardt and colleagues [10] completed a biomechanical study comparing two common surgical repair techniques. They evaluated the ultimate failure load and failure mechanism of an intact hamstring tendon compared to knotless and knotted anchor configurations for hamstring repair. The all-knotless suture anchor constructs failed at the highest maximal load of the 3 groups (767.18 ± 93.50 N), including the intact tendon group (750.58 ± 172.22 N). There was a statistically significant difference in load to failure when the all-knotless construct was compared with the all-knotted technique (549.56 ± 20.74 N). This demonstrated superiority of the knotless suture construct. The cadaveric study used a four suture-anchor configuration double row construct that was similar to the technique utilized in our case report. The proposed mechanism for this success is that the knotless design allows for reproducible equalization of pressure without relying on the variability of knot tying.

A double row technique allows for widely disrupted footprint pressure in efforts to improve contact with the bone tendon interface. Biomechanical studies evaluating double row constructs for rotator cuff repair have demonstrated less gap formation and improved tensile strength compared to single row repair [11]. Although, clinical studies have shown differing results on rerupture rates and outcomes following single or double row rotator cuff repair. Double row repair provides increased tendon-to-bone interface and ultimately increased healing potential. The double row construct allows the performing surgeon to set appropriate compression and overall hamstring tension.

Outcomes for operative repair of hamstring avulsions have shown high patient satisfaction; however, decreased strength, residual pain, and decreased activity level have been reported [9]. A cohort study performed by Shambaugh et al. [12] showed that patients can expect to regain approximately 90% strength of the uninjured extremity with acute hamstring repair using 2-4 suture anchors. Bowman et al. [13] found an overall satisfaction level of 94% in 58 patients treated with open or endoscopic suture anchor fixation, but runners were less satisfied. Overall, the majority of athletes (88%) returned to sports at 7.6 months, on average, with 72% returning at the same level. In comparison, the runners in the study returned to the same level only 50% of the time. Continued future research into outcomes is warranted.

Complications associated with operative HMC repair were reported as 23.17% by a recent meta-analysis, 7.99% being neurologic [14]. Acute repairs were found to have better outcomes compared to chronic repairs. Studies have shown more difficult exposure as well as more sciatic nerve neurolyses were performed in surgically treated chronic tears. The intimate relationship of the sciatic nerve and hamstring attachment at the ischial tuberosity makes it vulnerable to injury both at the time of hamstring avulsion and at time of operative repair [15]. Careful dissection, identifying, and protecting the sciatic nerve as well as acute repair help reduce the risk of iatrogenic injury [8]. In chronic cases, where distal tendon retraction is of concern, a longitudinal incision may be used to provide adequate exposure to allow for tendon mobilization and proper protection of the neurovascular bundle. Cosmetic concerns regarding incisional scar should be discussed with the patient during the informed consent process.

Marked variability exists for the postoperative physical therapy following hamstring repair owing to the various surgical techniques utilized. In a cross-sectional study of 50 rehabilitation protocols, 34% prescribed knee bracing, 23% prescribed hip bracing, and 14% did not specify the type of brace recommended. As for weight bearing, 40% of protocols advised non-weight bearing and 46% allowed toe-touch weight bearing. Advancement to full weight bearing was allowed at a mean of 7.1 weeks [16]. Gerhardt eliminated postoperative bracing and allowed his patients, following knotless suture anchor construct fixation, to eliminate weight bearing assistance at two weeks [10]. Toe-touch weight bearing with a knee brace flexed to 40 degrees for 4 weeks is recommended by Arner et al. to decrease the tension on the repair and is less cumbersome than a hip brace [1]. Arner's protocol included brace discontinuation at 4 weeks postoperatively with initiation of pain free arch of motion against gravity. Progressive return to sport protocol is recommended over a 6-month period. In this case vignette, postoperative care after a suture bridge technique included hinged knee brace for the first 6 weeks to control progressively increased range of motion.

## 5. Conclusion

The ultimate goals of proximal hamstring repair are to provide stable maximum strength fixation, optimizing bone-to-tendon healing potential, and to allow for early motion and physical therapy. Accelerated rehabilitation protocols may be initiated given the fixation strength of the all-knotless repair double row suture bridge repair technique. Our case demonstrates clinical applicability of a reproducible biomechanically strong construct that allows for early motion progression.

## Data Availability

All data available within case report manuscript (case report, single patient).

## Supplementary Materials

Supplementary Materials Video 1: displaying the clinical exam and surgical procedure of our clinical vignette (https://www.youtube.com/watch?v=ZQCkuINOeA4).
